# Improving risk communication: a proof-of-concept randomised control trial assessing the impact of visual aids for neurosurgical consent

**DOI:** 10.3389/fsurg.2024.1361040

**Published:** 2024-02-21

**Authors:** Despoina Chatzopoulou, Arif Hanafi Bin Jalal, Danail Stoyanov, Hani J. Marcus, Anand S. Pandit

**Affiliations:** ^1^ Department of General Surgery, Southampton General Hospital, Southampton, United Kingdom; ^2^ UCL Medical School, University College London, London, United Kingdom; ^3^ Wellcome/EPSRC Centre for Surgical and Interventional Sciences (WEISS), University College London, London, United Kingdom; ^4^ Victor Horsley Department of Neurosurgery, National Hospital for Neurology and Neurosurgery, London, United Kingdom; ^5^ High-Dimensional Neurology, Queen Square Institute of Neurology, University College London, London, United Kingdom

**Keywords:** informed consent, patient communication aids, health literacy, visual aids, health numeracy

## Abstract

**Introduction:**

Informed consent is a fundamental component in the work-up for surgical procedures. Statistical risk information pertaining to a procedure is by nature probabilistic and challenging to communicate, especially to those with poor numerical literacy. Visual aids and audio/video tools have previously been shown to improve patients' understanding of statistical information. In this study, we aimed to explore the impact of different methods of risk communication in healthy participants randomized to either undergo the consent process with visual aids or the standard consent process for lumbar puncture.

**Material and methods:**

Healthy individuals above 18 years old were eligible. The exclusion criteria were prior experience of the procedure or relevant medical knowledge, lack of capacity to consent, underlying cognitive impairment and hospitalised individuals. After randomisation, both groups received identical medical information about the procedure of a lumbar puncture in a hypothetical clinical scenario via different means of consent. The control group underwent the standard consent process in current clinical practice (Consent Form 1 without any illustrative examples), whereas the intervention group received additional anatomy diagrams, the Paling Palette and the Paling perspective scale. Anonymised questionnaires were received to evaluate their perception of the procedure and its associated risks.

**Results:**

Fifty-two individuals were eligible without statistically significant differences in age, sex, professional status and the familiarity of the procedure. Visual aids were noted to improve the confidence of participants to describe the risks by themselves (*p* = 0.009) and participants in the intervention group felt significantly less overwhelmed with medical information (*p* = 0.028). The enhanced consent process was found to be significantly more acceptable by participants (*p* = 0.03). There was a trend towards greater appropriateness (*p* = 0.06) and it appeared to have “good” usability (median SUS = 76.4), although this also did not reach statistical significance (*p* = 0.06)

**Conclusion:**

Visual aids could be an appropriate alternative method for medical consent without being inferior regarding the understanding of the procedure, its risks and its benefits. Future studies could possibly compare or incorporate multiple interventions to determine the most effective tools in a larger scale of population including patients as well as healthy individuals.

## Introduction

Informed consent is a fundamental component in the work-up for surgical procedures. Statistical risk information pertaining to a procedure is by nature probabilistic and challenging to communicate, especially to those with poor numerical literacy (1). In the context of health literacy, numeracy refers to a person's ability to understand and interpret clinical and public health data (1). This is considered to be low in the general population with most adults having difficulty in converting small frequencies such as “1 in 1,000” to 0.1% (2).

In addition to individual patient factors, risk perception is affected by different formats of presentation resulting in “framing bias” (3, 4). This phenomenon refers to variable decision outcomes depending on the different modes of presentation of identical data among individuals. Healthcare professionals may communicate statistical risk information using descriptive terms such as “uncommon” or “rare” or as percentages and proportions. Risks are often verbally explained prior to obtaining consent without the acknowledgement of patients' numerical skills and without the use of other aids. In neurosurgery, patient recall of risks without their associated probabilities is noted to be poor with less than 50% of risks being retained (5, 6). This percentage further decreases over time following the original consent process and post-operatively (6). Ensuring patients have an adequate understanding of statistical risk is vital to prevent potential litigation with “lack of informed consent” being a factor in up to 40% of neurosurgical medicolegal cases (7).

The impact of several decision aids on patients' overall understanding of surgical or interventional procedures has been studied (8–11). However few studies compare multiple formats concurrently (9, 12) while focusing on patient understanding of statistical risk. Visual aids and audio/video tools have been shown to improve patients' understanding of statistical information (8, 9, 13, 14). Our study demonstrates the use of visual statistical risk communication adjuncts in a simulated procedural consent of a lumbar puncture and presents implementation outcome measures. We analyse participants’ understanding of the procedure, but also more specifically, their understanding of procedural complications, associated numerical probability and attitudes related to surgical consent.

## Methods

### Participants

Inclusion criteria were healthy individuals above 18 years old not requiring a lumbar puncture and without any underlying cognitive impairment. Exclusion criteria were individuals with prior experience receiving, performing or observing the procedure, individuals lacking capacity to consent and hospitalised individuals. The educational background of the participants varied from secondary level of education to postdoctoral level of studies within and outside the medical field.

### Video generation

**Figure 1:**
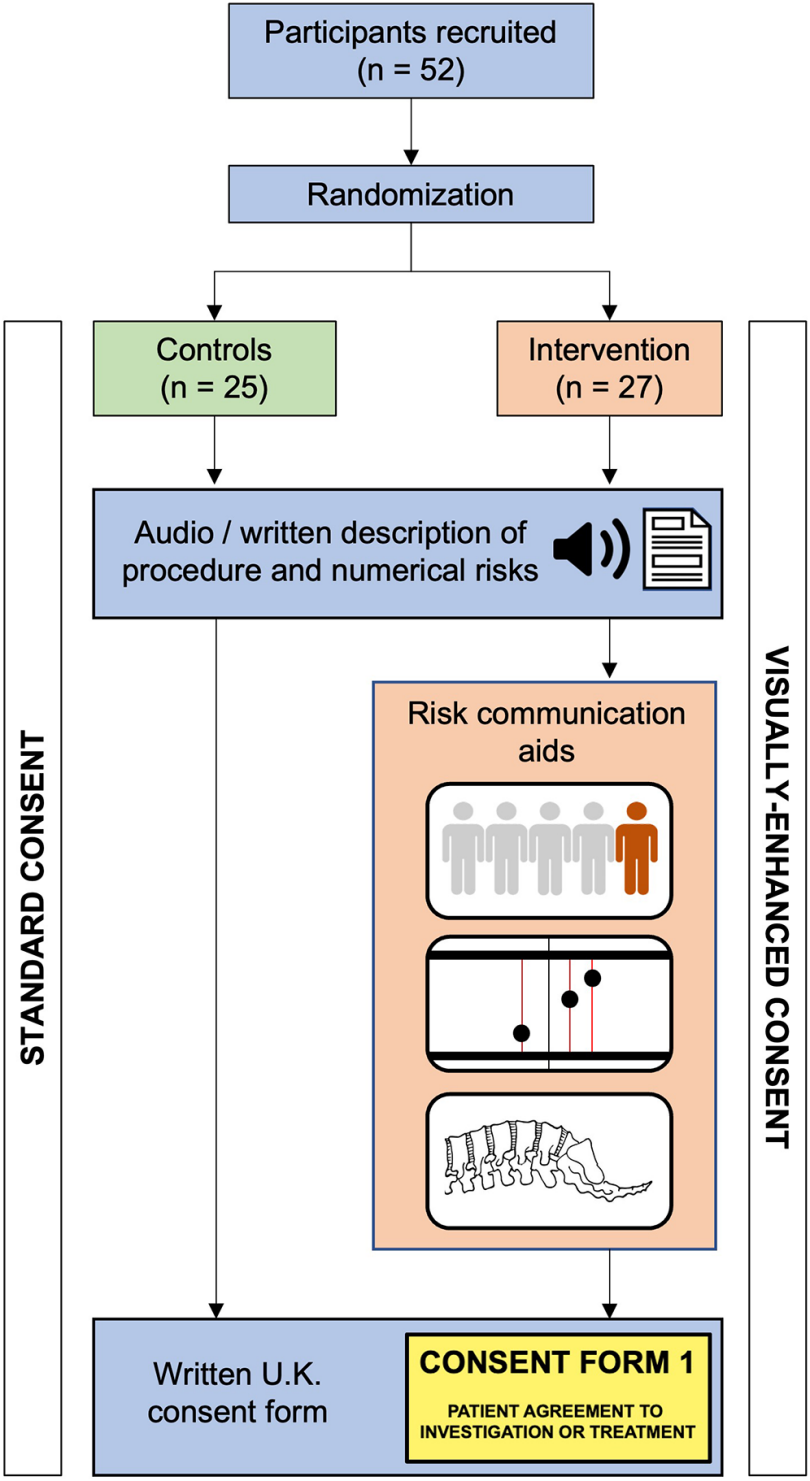
Flow diagram of participants allocated to the intervention (visually-enhanced consent) or control group.

**Figure 2:**
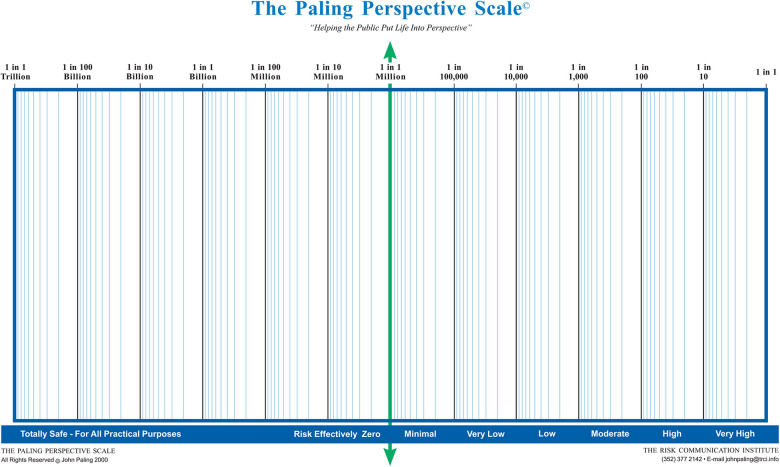
The paling perspective scale. Reproduced with permission from The Risk Communication Institute.

**Figure 3:**
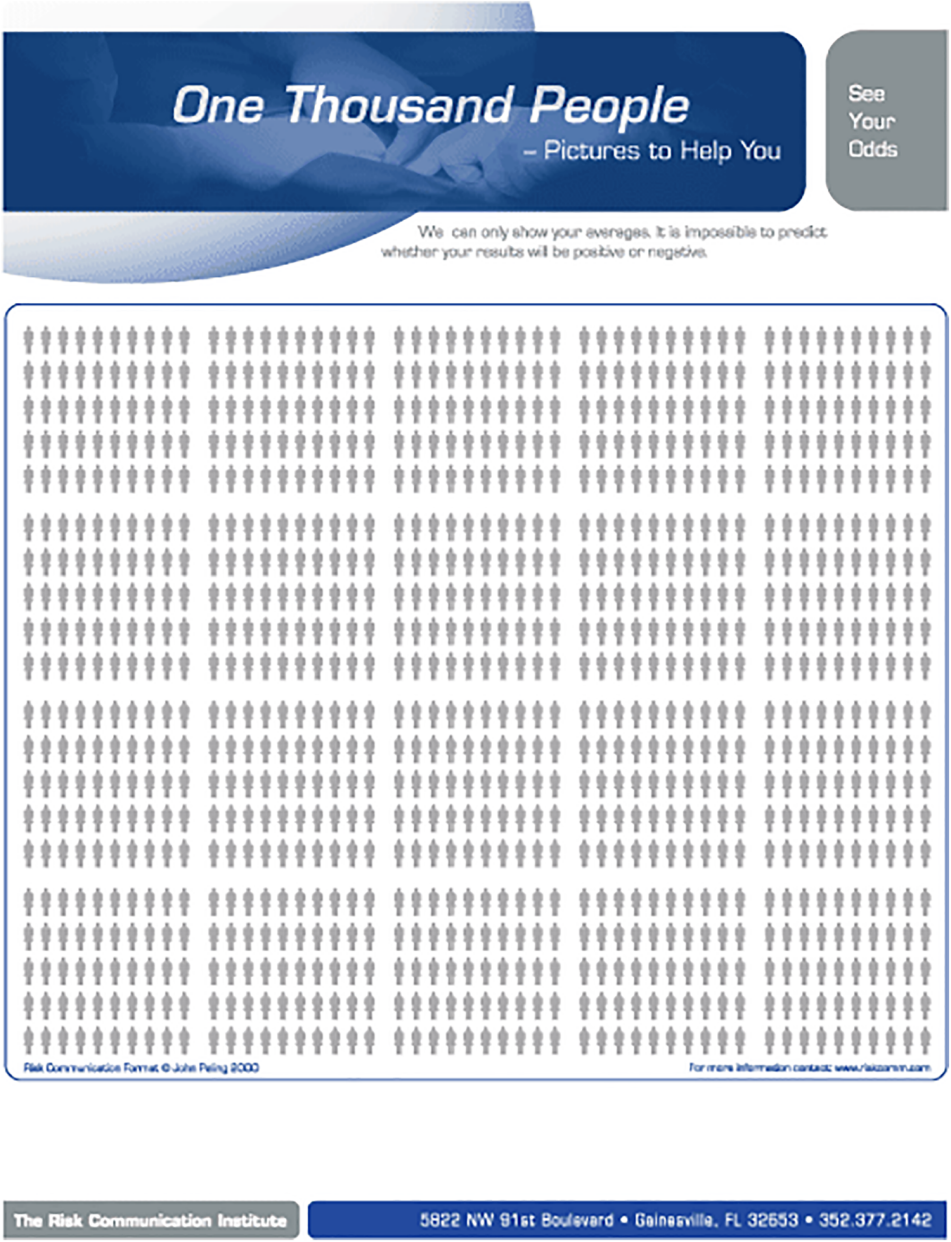
One thousand people figure. Reproduced with permission from The Risk Communication Institute.

The video recording was made with the use of Vyond (Vyond, San Mateo, Ca), a cloud-based video animation tool. The same voice recording was used in both groups, ensuring the content of the medical information is identical in both groups. While both groups received identical medical information and content regarding hypothetical clinical scenarios in which an intervention was consented for, the means of consent differed (Figure 1). The control group had the same risks verbally explained without any aids as is typical for pre-operative surgical consent. The intervention group received the statistical information in the form of visual aids—anatomy diagrams and Paling scales (Figures 2, 3) (15). At the end of the videos, the participants of both groups were presented with a written consent form as it is used in clinical practice summarising the name, the risks and the benefits of the procedure. It was requested that participants sign the consent form as they would do in clinical practice.

Please find the relevant video links below:

• **Group A (Control Group):** https://youtu.be/-QuOp-BTEKM”\h
• **Group B (Visual Aid Group):** https://youtu.be/wFe4KXEq8hw”\h

### Questionnaire generation

Participant knowledge and feedback with the respective consent process were assessed using a 41-item questionnaire developed in Qualtrics (Qualtrics, Provo, UT), the different sections and their respective questions are detailed in Table 1. The first section consisted of demographic information including age, gender and familiarity with the procedure. The second section required participants to rate their agreement to a series of seven statements specifically written for the study using a 5-point Likert scale. The intervention group received an additional three statements relating to the helpfulness of the three visual aids used in the video (Anatomy diagram, ten-man diagram and relative risk diagram). The ten-man diagram and the relative risk diagram are based on the Paling diagram and Paling Palette respectively with the permission of the Risk Communication Institute (Figures 2, 3) (15). A free-text prompt asking for additional feedback to improve the consent process was also included. This was followed by seven multiple choice questions assessing participants' knowledge on the procedure. The final section consisted of 19 questions to assess the implementation of the process in clinical practice using four validated scales (16, 17).

**Table 1:** Questions included in the questionnaire relating to knowledge of the procedure, participant sentiment, usability, acceptability and appropriateness.

Validated scale	Description	Questions included
Procedure related questions	This section was composed of seven Likert scale statements (“Strongly disagree” to “Strongly agree) and was used to assess participant's feelings towards their respective consent processes. The following statements were designed specifically for this study.	1. I felt the procedure was clearly explained 2. I understand the benefits of the procedure 3. I understand the risks of the procedure 4. I feel my expectations are well managed 5. I would be able to explain the risks of the procedure to someone else 6. I feel overwhelmed with medical information
Knowledge assessment	Questions were based on the information provided to both groups in each video and used to assess participant knowledge/recall of the procedure and its associated risks. The answers to each question were present in both videos.	1. The needle for lumbar puncture is inserted in the a. Spinal cord b. Spinal canal c. Subcutaneous tissue 2. The most common risk of lumbar puncture is a. Headache b. Back pain c. Bleeding d. Infection 3. What is the risk of infection to skin, brain or spinal cord? a. More than 1 in 10,000 b. Equal to 1 in 10,000 c. Less than 1 in 10,000 4. What is the risk of a headache following a lumbar puncture? a. 10% b. 30% c. 40% 5. What is the risk of back pain following a lumbar puncture? a. 10% b. 15% c. 20% 6. What is the lifetime risk of death in the UK from a road traffic accident? a. Approximately 42 in 10,000 b. Approximately 48 in 10,000 c. Approximately 52 in 10,000 7. What is the risk of bleeding following a lumbar puncture? a. Less than 1% b. Less than 2% c. Less than 5%
The system usability score (SUS)^2^	A reliable tool for measuring the usability of a system, a product or a method. It consists of a 10 item questionnaire, where the individuals provide responses ranging from “Strongly Agree” to “Strongly Disagree”.	1) I think that, if I needed to, I would like to use this consent process 2) I found the consent process unnecessarily complex 3) I think that the way the risk was presented for this consent process was appealing 4) I think that I would need the support of a technical person to be able to use the consent process 5) I found the various functions in the consent process were well integrated 6) I thought there was too much inconsistency in the consent process 7) I would imagine that most people would learn to use this consent process very quickly 8) I found the consent process very cumbersome of use 9) I felt very confident using this consent process 10) I needed to learn a lot of things before I could get going with this consent process
Acceptability of intervention measure (AIM)^3^	Acceptability is the perception among individuals that a given method is agreeable and satisfactory. A Scale was created for each group and the average score of the following responses was recorded. The value of each response ranged from 1 to 5.	1) This consent process meets my approval 2) This consent process is appealing to me 3) I like this consent process 4) I welcome this consent process
Intervention appropriateness measure (IAM)^3^	Appropriateness is the perceived relevance and compatibility of the proposed method for consent among participants. A Scale was created for each group and the average score of the following responses was recorded. The value of each response ranged from 1 to 5.	1) This consent process is fitting 2) This consent process is suitable 3) This consent process is applicable 4) This consent process seems like a good match

### Participant recruitment

The study was advertised online via social media and mailing lists within our institutional academic community therefore we are not able to report the number of individuals approached for the study. Furthermore, given the anonymised nature of the study it is not known what proportion of individuals directly approached by study staff completed the survey. Upon clicking the link to the questionnaire, participants were randomised in a 1:1 ratio using Qualtrics' built in randomization software to receive either the control group with the standard informed consent process or the intervention group which featured various visual aids. Researchers were blinded to the assignment of individual participants to the respective groups. Participant recruitment took place over a two-month period from 27th March 2022 to 26th May 2022. Data collection was concluded when the target sample size was reached. Participants completed anonymised questionnaires relating to how they recalled and perceived statistical risks, on their understanding of the procedure and how usable, acceptable, and appropriate their consent method was, using validated scales (17).

### Statistical analysis

A sample size calculation found a minimum of 25 participants in each group was required for a significant difference of 0.4 points between groups based on a 5-point Likert scale (Cohen's *d* = 0.8, alpha = 0.05, power = 0.80, GPower v = 3.1). All statistical analysis was performed in RStudio (RStudio Team, Boston, MA). Normality of the data was assessed using the Kolmogorov–Smirnov test, and by visually inspecting the distribution. If data was non-parametric, the Mann–Whitney *U*-test was used to assess the differences between the control and intervention groups, with a *p*-value < 0.05 being considered as significant.

### Ethical approval

This study's protocol was reviewed and approved by an institutional ethics committee (UCL Research Ethics Committee Project ID: 21837/001). Prior to being able to complete the questionnaire, participants were asked to confirm they meet the inclusion criteria of the study and voluntarily consent to completing the study through Qualtrics. Participants were directed to a participant information sheet within the study. Names and contact information of participants was not collected. Our study was retrospectively registered on ClinicalTrial.gov on 8th February 2023 (NCT05717465).

## Results

52 participants were included in the study. The demographics of the eligible population are presented after the randomisation, with reference to the age, sex, professional status, understanding of, and prior familiarity with the procedure between groups (Table 2). There was no statistical difference in numerical risk recall, and those in the intervention (visually enhanced consent) group were not inferior in their subjective understanding of the procedural benefits (*p* = 0.29) (Table 3). However, the intervention group seemed to have a better understanding of the risks (*p* = 0.05), they thought they could better explain the risks to others (*p* = 0.01), and they seemed to feel less overwhelmed with information (*p* = 0.03). Furthermore, the enhanced consent process was found by participants to be significantly more acceptable (*p* = 0.03). It showed a trend towards greater appropriateness (*p* = 0.06) and it appeared to have “good” usability (median SUS = 76.4), although this also did not reach statistical significance (*p* = 0.06).

**Table 2:** Table of demographics between control group and visual aids group.

	Visual aid group (*n* = 25)	Control group (*n* = 27)
Sex		
Male	12	11
Female	12	16
Prefer not to say	1	0
Age		
Age (median and age range)	23 (19–56)	22 (19–28)
Professional status		
Student on another course	10	12
Medical student	6	10
Professional in non-healthcare related field	9	5
Completed education		
Secondary school	5	4
Diploma/College certificate	3	3
Bachelor's/Master's degree	12	20
Doctoral/PhD degree or higher	5	0
Median familiarity with procedure	1.96	1.96
Method of completion		
Laptop	18	16
Mobile device (phone, tablet)	7	11

**Table 3:** Outcomes by consent method.

		Control group median (IQR)	Visual aid group median (IQR)	Control group mean (SD)	Visual aid group mean (SD)	Mean difference	*p*-value (MWU test)
Procedure related statements	I felt the procedure was clearly explained.	4.00 (4–5)	4.00 (4–5)	4.04 (0.89)	4.30 (0.72)	0.26	0.33
	I feel my expectations are well managed.	4.00 (3–4)	4.00 (3.5–5)	3.80 (0.87)	4.15 (0.91)	0.35	0.15
	I understand the benefits of the procedure	4.00 (3–5)	5.00 (4–5)	3.88 (1.17)	4.26 (0.90)	0.38	0.26
	I understand the risks of the procedure.	5.00 (4–5)	5.00 (5–5)	4.56 (0.51)	4.82 (0.40)	0.26	0.05
	I would be able to explain the risks of the procedure to someone else.	4.00 (3–4)	5.00 (4–5)	3.88 (0.78)	4.44 (0.75)	0.56	0.01
	I feel overwhelmed with medical information.	2.00 (1–3)	2.00 (1–2)	2.40 (1.15)	1.70 (0.72)	−0.70	0.03
Objective assessment	Risk recall score	6.00 (6–7)	6.00 (5.5–7)	6.12 (1.17)	5.89 (1.37)	−0.23	0.68
	AIM	3.25 (3–4)	4.0 (3.38–4.75)	3.41 (0.95)	3.97 (0.88)	0.56	0.02
	IAM	3.63 (3.25–4.13)	4.00 (3.5–4.75)	3.56 (0.83)	4.00 (0.84)	0.44	0.06
	SUS	70.0 (60–80)	75.0 (68.75–88.75)	69.10 (13.27)	76.48 (13.89)	7.38	0.06

MWU, Mann–Whitney *U*-test, IQR, interquartile range, AIM, acceptability of intervention measure, IAM, intervention appropriateness measure, SUS, system usability scale.

## Discussion

Our study suggests that that visual risk communication adjuncts may offer some advantages when compared to traditionally obtained surgical consent, particularly with reference to subjective understanding and attitudes toward procedural risks. Statistically significant improvements were noted in the trial group regarding the ability to explain risks to others and greater acceptability and good usability of the consent adjunct, whilst also feeling less overloaded with medical information.

We acknowledge limitations in our approach, including the choice of individuals and the intervention. Recruiting healthy adults allowed us to test our hypothesis in a controlled simulated setting without bioethical concerns, but this does not fully replicate the atmosphere and anxieties associated with consent in hospital, nor having the procedure performed in the context of experiencing a disease process. The interventional choice of a lumbar puncture although simple remains a procedure that requires written informed consent and carries non-trivial complications (18). Further work should assess procedures of greater complexity, and the impact of framing bias, namely, whether consent outcomes depend on the mode and the conditions of presentation (3, 19). Another limitation of the study is the overall high rate of literacy in both groups according to the table of demographics. In this case, the numerical literacy of the participants could be considered higher than expected in the general surgical population and therefore not easily generalisable.

This controlled trial evaluates the utility of risk communication adjuncts for surgical consent and contributes to this expanding field of bioethics. The surgical decision-making process is not only related to risk perception, but also to risk acceptance in accordance with an individual's threshold for specific postoperative complications and the frequency in which they occur (20). To make consent fully informed, patient decision-making should be in line with, or adapted to, their probabilistic understanding of the intervention. Considering the findings of our study, we believe that visual risk communication tools could enable a more informed consent process. The systematic application of validated and widely accepted psychometric tools to evaluate patients' perception of a procedure and its associated risks in the field of medical consent is required to collect further supporting evidence. This proof-of-concept study provides a suggested methodology that could be useful in future larger-scale patient populations studies to translate our conclusions into clinical practice.

## Data Availability

The raw data supporting the conclusions of this article will be made available by the authors, without undue reservation.
